# United States National Healthcare Policies 2015: An Analysis with Implications for the Future of Medicine

**DOI:** 10.7759/cureus.451

**Published:** 2016-01-07

**Authors:** Harjus S Birk

**Affiliations:** 1 Neurological Surgery, UCSF School of Medicine, Howard Hughes Medical Institute Research Fellow

**Keywords:** healthcare policy, healthcare expenditures, Affordable Care Act, quality of care, affordability

## Abstract

There is little doubt that the tenure of President Barack Obama and implementation of the Affordable Care Act has had a profound effect on the United States healthcare delivery system in terms of the organization, finances, and clinical aspects of medical practice. As we enter the 2016 presidential election, looming issues of health affairs include 1) Is affordability achievable and can it be achieved without sacrificing the physician-patient relationship? and 2) Does practice consolidation and control by insurance providers cast physicians in a role as technicians?

In countries such as the United Kingdom, policies seeking to increase healthcare affordability without sacrificing the quality of care have been implemented, as manifested through not only socialized medicine but also a general goal of cost cutting without sacrificing patient care. In addition, although done more as a tactical move with little impact on the overall budget, the healthcare benefits of political leaders in the United Kingdom are being trimmed in order to increase citizen buy-in in the healthcare model. This article compares recent healthcare policy changes in the United States to those of some constitutional democracies. The attitudes of healthcare stakeholders, including patients, physicians, and political leaders, are also analyzed. It is argued that the evolution of health affairs internationally is driven largely by efficacious political and economic factors, and that it behooves United States healthcare policy makers to note the impact of these international changes and to integrate the necessary changes in order to enhance patient care.

## Introduction and background

Before comparing the healthcare policies of countries, such as the United Kingdom, to that of the United States, it is first necessary to analyze the current effects of the United States healthcare system on its medical professionals. Namely, physicians in solo practice as well as in groups have become employees of non-physician controlled healthcare delivery organizations, and this development has exerted impactful influence on all phases and sectors of medicine [1-2]. One striking effect is evidenced by the selection of medical specialties by young physicians. Institutional and large group practices have become increasingly attractive to young physicians, especially those who have incurred significant debt in order to complete their medical education. As opposed to private practice, it eases many of the burdens of finance, regulations, and retirement benefits [3].

The control or outright ownership of hospitals and similar medical institutions is also a major development in the recent tenure of President Barack Obama. Previously, both not-for-profit and for-profit organizations operated hospitals in a cooperative manner in which the for-profit entities offered medical practices which were profitable and thus helped financially support the not-for-profit entities. Today, however, the number of not-for-profit institutions has rapidly diminished as corporate entities assume control to reap the clear financial benefits of for-profit organizations. In 2009, nearly 67% of all United States urban hospitals were nonprofit; interestingly, today this number has dwindled to less than 50% [4]. This shift has taken place as for-profit hospitals are believed to collect more revenue per each patient admission than do not-for-profit hospitals, although certain percentages have not been reported. A recently published study assessed the overall profitability of nonprofit and for-profit hospitals, taking into consideration different medical services that may be offered among each [5]. The results indicated that for-profit institutions were more likely to obtain higher profits from the same medical services as compared to nonprofit institutions. To ensure that hospital ownership status contributed to these results rather than geography, several sensitivity tests were performed that further confirmed the above findings. Ultimately, the absorption of medical practices and, on occasion, the practices of an entire community, has grown and has had a major impact on the lives of heretofore-independent physicians.

## Review

In the setting of medical development, the power controlling the cost of healthcare is shared by insurance companies, hospitals, and pharmaceutical companies alike. The United States Department of Health and Human Services, as well as the Joint Commission on Accreditation of Hospital Organizations, are involved in overseeing these stakeholders. Many hospitals have answered in part to the rising financial burdens in the United States by hiring non-physician executives to serve as hospital administrators because they demand less salary than physicians [6-7]. A recent review article analyzed the role physician executives, as well as non-physician executives, play when operating as Chief Executive Officers of hospitals. It reported that hiring non-medical professionals to serve as executives is often preferred over hiring healthcare professionals with medical degrees because the former demand less salary [8]. It is unlikely that a shift in hospital power among non-physician executives will occur because the political power and sheer large numbers of these executives may create a barrier difficult for physicians to surpass. However, it is important to note that even though one might expect that takeover of management by non-physicians would result in lower costs because of greater efficiencies, this may not necessarily be the case because over time the executives may demand increased salaries [9-10]. 

On a national scale, there is a large absence of central standardized control of the fees charged by health care professionals in both United States hospitals and private practice settings. As a result, prices have steadily increased for diagnostic tests, medical appointments, and similar services among similarly geographic distributions [11-12]. It is important to note that some control mechanisms do exist to determine physician reimbursement as compared to what the physicians charge that patient. For instance, Medicare uses a system termed "relative value unit-based productivity" to decide how much it will reimburse physicians for services and procedures. For the purpose of this article, we focus on the prices charged by the healthcare provider because the actual cost to the provider and reimbursement obtained are challenging to procure.

In 2012, the physician price for a private practice gynecologist to perform a laparoscopic hysterectomy in Sacramento was $47,500 as compared to $34,400 charged by a gynecologist in Orange County. Since these statistics were obtained from private practice, the difference in the laparoscopic hysterectomy may not seem out of the ordinary. However, this cost disparity becomes even more explicit when considering that, when comparing hospital prices, a patient seeking a knee replacement operation at Sutter General Hospital was charged $86,002 in 2010 as compared to $126,292 for the same procedure at the UC Davis Medical Center only a few miles away [13]. Confounding variables that may in part explain price differences for medical services include geography and whether the hospital is a teaching or non-teaching institution because some areas with higher costs of living may be subject to similar medical cost adjustments. Nevertheless, when analyzing the prices charged for procedures by hospitals and private practices in similar cities in the same state, the difference in price becomes apparent. Although the actual service cost as well as reimbursement to the physician or hospital likely varies from the price charged for the service, it is evident that patient care has variable prices. 

There has been some effort by Congress to exert standardized controls aimed to ensure the cost of United States healthcare does not skyrocket and that the quality of medical care is not curtailed. The Affordable Care Act has a provision whereby hospitals are fined up to 3% of their Medicare payments if a certain quantity of patients return to the hospital within 30 days of being discharged, with the reasoning that these readmissions are indicative of poor care. In 2015 alone, 2,592 hospitals in the United States suffered from readmission penalties that totaled $420 million [14]. Although this provision may be reasonable in many cases, in others it may be unavoidable, such as in the case of a hypothetical homeless man with pneumonia who does not take his antibiotics upon discharge and therefore returns the emergency department within the 30 day period. Although provisions such as this are a step in the right direction, the ideal goal would be one that does not punish hospitals for unavoidable patient cases, such as the aforementioned. It has been argued that one step towards achieving this goal would be for members of Congress to refrain from obtaining very generous and unique healthcare plans [15]. For instance, members of Congress qualify for medical benefits that ordinary federal workers do not and are often able to receive medical services from the Office of the Attending Physician of the United States Capitol [16-17]. This makes it challenging for Congress to relate to and act upon the need for cost-control that most Americans seek when pursuing comprehensive and holistic medical care. In order to alleviate this issue, it may prove valuable to follow the precedent set by the United Kingdom of trimming the health benefits of its political leaders. Although this may have a relatively small effect on decreasing budget deficits, it may most importantly increase the faith of the people in the healthcare system and in the political leaders who are molding these systems. This may be a logical step towards bridging the gap between Congress and the American people as both groups will be operating under similar medical care plans. Given the needs of the American people, a greater effort must be made targeting a holistic and financially accessible national healthcare protection. 

It is necessary to note that the organizations comprising that industry cannot exist without an adequate source of funding. The cost of pharmaceutical products must in some fashion reflect the expenses incurred in research as well as promotion and sales. For instance, drug development in the United States in 2011 exceeded $1 billion and has been steadily rising each year since [18]. With increasing pressures to improve medical care through revolutionary advancements and therapeutic agents, the expenditures of the pharmaceutical companies are understandably high. The burden of cost becomes an issue when considering that local, state, and national regulations add to product cost for consumers. The medication component, essential as it is, exerts significant upward pressure on the cost of medical care for physicians and patients. In 2014, 52% of United States doctors proclaimed that their medical staff spends too much time communicating with insurance companies to approve lofty and complex medication requests [19]. In the future, it may prove invaluable to ascertain a similar statistic for the United Kingdom in order to compare efficiencies of the varying health care systems.

In communities throughout the United States, whether urban or rural, hospitals are starting to buy physician practices. According to the American Hospital Association, between 2000 and 2010, there was a 32% increase in the number of previously private practice physicians who transitioned to become employees of local hospitals. Absorption of physician practices does have advantages for physicians in minimizing financial responsibilities and the numerous bureaucratic measures that have become part of the practice of medicine, such as nuanced payment methodologies and administrative tensions [20-21]. An income guarantee within the hospital infrastructure, even though perhaps lower than the practicing physician has been accustomed to in private practice, together with diminished pressures of the ever increasing business aspects of medicine, make for a more appealing lifestyle as a hospitalist. Moreover, the growth of closed physician panels and groups makes it more challenging for the young physician to build a practice. In essence, the hospitals exert control over not only inpatient care but also over a significant portion of outpatient care. In that setting, the hospitals have also absorbed the physician-insurance link. The unfortunate result of this is that the interests of the physicians become secondary to the interchange and negotiations between insurance companies and the hospitals.

It is likely that the country and its inhabitants will be better off economically when healthcare expenditures are controlled, whether through a socialized healthcare mechanism, as evidenced by the United Kingdom, or through simple cost-cutting. Although perhaps counterintuitive, neither studies nor associations reveal that by spending more on medical care, people will be healthier. In fact, data indicates that improved health results can be more readily observed in countries, such as France and Amsterdam, with a lower per capita outlay for healthcare than the United States [22]. Fifty-nine percent of physicians in the United States report that their patients struggle to pay for medical treatment and that this inability to adequately finance healthcare negatively impacts the patients. In stark comparison, only 4% of Norway physicians and 13% of United Kingdom physicians reported that affordability was a concern for their patient population [23]. It is important to note that controlling healthcare expenses in the United States will not automatically result in the benefits seen in the aforementioned European countries, but over time, a more holistic healthcare model that equalizes the power between political leaders, hospitals, and executives will inevitably lead to a more patient-focused healthcare system, which places patient safety at paramount importance.

## Conclusions

With the upcoming 2016 Presidential Election, it is explicit that the next several years will foster significant changes in the health policy affairs of the United States The trend toward larger institutions controlled by business executives and geared towards generating financial gains will continue to grow. This may increase the control over the practice of medicine, which may not keep in alignment with the philosophy of physicians and other healthcare professionals. Fortunately, national legislation has made it possible to expand the availability of medical services across the country and across all layers of society. 

In due course, adjustments should be made in order to introduce financial adjustments that will shift funds from non-clinical to clinical areas of medicine, such that the quality of patient care is not sacrificed. Experience in other areas of the world, such as the United Kingdom, has revealed that medical care benefits are invaluable towards promoting and maintaining holistic and comprehensive healthcare. 

## References

[1] Hall MA, Lord R (2014). Obamacare: What the Affordable Care Act means for patients and physicians. BMJ.

[2] Dunn A, Shapiro AH (2015). Physician payments under health care reform. J Health Econ.

[3] Farber M, Cheng A, Cuff A (2007). Hospital and private practice partnerships: Which model is right for you?. J Oncol Pract.

[4] Horwitz J (2005). Making profits and providing care: comparing nonprofit, for-profit, and government hospitals. Health Aff (Millwood).

[5] Horwitz JR, Nichols A (2009). Hospital ownership and medical services: Market mix, spillover effects, and nonprofit objectives. J Health Econ.

[6] Eagle C (2016). Physicians at the executive table. Healthc Manage Forum.

[7] Brislen H, Dunn A, Parada A, Rendon P (2015). Addressing the primary care shortage on a shoestring: a successful track in an internal medicine residency. Acad Med.

[8] (2015). Health law impacts primary care doctor shortage. http://www.stltoday.com/news/special-reports/mohealth/health-law-impacts-primary-care-doctor-shortage/article_c1f4d681-11d6-5d34-abc3-ddf64f2193c6.html.

[9] (2015). A doctor shortage and the health law. http://www.nytimes.com/2012/08/02/opinion/a-doctor-shortage-and-the-health-law.html.

[10] (2015). The 2008 health confidence survey: rising costs continue to change the way americans use the health care system. The.

[11] Falcone RE, Satiani B (2008). Physician as hospital chief executive officer. Vasc Endovascular Surg.

[12] (2015). Generic drug makers see a drought ahead. http://www.nytimes.com/2012/12/04/business/generic-drug-makers-facing-squeeze-on-revenue.html.

[13] van der Reis L, Xiao Q, Savage G (2007). A retrospective on access to health care. Int J Health Care Qual Assur.

[14] (2015). Is Obamacare punishing hospitals the wrong way?. http://www.huffingtonpost.com/entry/obamacare-hospital-readmissions_55f732e1e4b0c2077efbb909.

[15] Hsia RY, Antwi YA, Weber E, Nath JB (2014). A cross-sectional analysis of variation in charges and prices across California for percutaneous coronary intervention. PLos One.

[16] (2015). The computer will see you now. http://www.nytimes.com/2009/03/06/opinion/06coben.html.

[17] (2015). Barack Obama and Joe Biden’s plan to lower health Care costs and ensure affordable, accessible health coverage for all. http://change.gov/agenda/health_care_agenda/.

[18] Matthews AW (2015). Same doctor visit, double the cost. The.

[19] Schoen C, Osborn R, Huynh PT, Doty M, Peugh J, Zapert K (2006). On the front lines of care: primary care doctors' office systems, experiences, and views in seven countries. Health Aff (Millwood).

[20] 18]. HIMSS Steering Committee (2015). HIMSS Steering Committee: Electronic health records, a global perspective. http://www.himss.org/ResourceLibrary/ResourceDetail.aspx?ItemNumber=7392.

[21] Kondro W (2012). Private practice on life support in America. CMAJ.

[22] Karman J, Sevil M (2015). 30,000 Amsterdam citizens hit through savings and care. Het.

[23] Aiken LH, Sermeus W, Van den Heede K, Sloane DM, Busse R, McKee M, Bruyneel L, Rafferty AM, Griffiths P, Moreno-Casbas MT, Tishelman C, Scott A, Brzostek T, Kinnunen J, Schwendimann R, Heinen M, Zikos D, Sjetne IS, Smith HL, Kutney-Lee A (2012). Patient safety, satisfaction, and quality of hospital care: cross sectional surveys of nurses and patients in 12 countries in Europe and the United States. BMJ.

